# Education moderates the effects of large central artery aging on cognitive performance in middle‐aged and older adults

**DOI:** 10.14814/phy2.14291

**Published:** 2019-12-12

**Authors:** Lyndsey E. DuBose, David J. Moser, Emily Harlynn, Jess G. Fiedorowicz, Gary L. Pierce

**Affiliations:** ^1^ Department of Health and Human Physiology University of Iowa Iowa City Iowa; ^2^ Department of Psychiatry University of Iowa Iowa City Iowa; ^3^ Department of Epidemiology University of Iowa Iowa City Iowa; ^4^ Department of Internal Medicine University of Iowa Iowa City Iowa; ^5^ Abboud Cardiovascular Research Center University of Iowa Iowa City Iowa

**Keywords:** aging, blood pressure, cognition, large elastic artery stiffness

## Abstract

Central artery aging, including elevated aortic stiffness, central blood pressure (BP), and pulse pressure (PP), is a novel risk factor for the development of age‐associated cognitive dysfunction. Individuals with higher educational attainment may develop greater brain pathology prior to the onset of cognitive decline. However, whether education moderates relations between central artery aging and cognitive performance is unknown. We hypothesized that years of formal education would moderate the relation between central artery aging and cognitive performance in middle‐aged/older (MA/O) adults (*n* = 113, age 67.3 ± 0.7 years). Significant interactions between education*central systolic BP (*β* = .21, *p* = .02) and education*central PP (*β* = .22, *p* = .01) demonstrated weaker associations between central BP and PP with processing speed performance in those with higher education. Similarly, education moderated the relation between aortic stiffness (carotid‐femoral pulse wave velocity, cfPWV) and executive function performance (*β* = .21, *p* = .02). To test if the relation between central arterial aging and cognitive performance was captured by a predetermined education threshold, MA/O adults were secondarily categorized as ≤high school (HS) (i.e., ≤12 years, *n* = 36) or >HS (≥13 years, *n* = 77). Higher central systolic BP was associated with slower processing speed (≤HS: *r* = −.59, *p* < .001 vs. >HS: *r* = −.25, *p* = .03) and weaker executive function (*r* = −.39, *p* = .03 vs. *r* = −.32, *p* = .006). Higher cfPWV was selectively correlated with weaker executive function performance (*r* = −.39, *p* = .03) in ≤HS only and this association significantly differed between education groups. Educational attainment appears to moderate the adverse effects of central artery aging on cognitive performance among MA/O adults.

## Graphical abstract

Studies investigating the link between central artery aging and cognitive decline commonly statistically adjust for educational attainment. Education moderates the detrimental effects of neuropathology to attenuate declines in cognition by increasing cognitive reserve. This study demonstrates for the first time that formal educational attainment moderates the relation between central artery aging and cognitive performance such that middle‐aged and older adults with lower educational attainment may be more sensitive to age‐related increases in central artery stiffness and blood pressure.

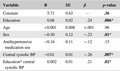

## 1 INTRODUCTION

Development of age‐related cognitive decline is a major cause of morbidity and mortality in the United States (Alzheimer’s Association, 2018). Healthcare costs associated with dementia are estimated to be 60% greater than costs related to cardiovascular disease (CVD) and cancer and are expected to rise as the prevalence of dementia surges from 5 million to 15 million by 2050. Increased attention has been directed at understanding the complex pathophysiology of cognitive decline and risk factors that contribute to or moderate the development of age‐related cognitive decline in order to guide the prevention and treatment. In this regard, modifiable risk factors for the development of cognitive decline include CVD risk factors (Cooper et al., 2016; Geijselaers et al., 2016; Hanon et al., 2005) such as elevated blood pressure (BP), diabetes, and increased large central elastic artery stiffness, as well as sociobehavioral risk factors such as educational attainment (Hankey, 2018; Mortamais et al., 2014). However, the mechanism by which cognitive impairment manifests and progresses likely results from complex interactions between modifiable and nonmodifiable risk factors across the lifespan.

Age‐related stiffening of the large central arteries, including the aorta and carotid arteries, is a novel risk factor for the development of cognitive impairment (Hughes et al., 2018; Mitchell et al., 2011; Pase et al., 2016). Large central elastic artery stiffness is an independent predictor of longitudinal changes in cognitive performance (Scuteri et al., 2007) among middle‐aged and older (MA/O) adults mediated in part through the development of neuropathology (Cooper et al., 2016). Stiffening of the aorta reduces the ability of the large central elastic arteries to buffer elevated central systolic BP and pulse pressure (PP) generated by left ventricular contraction and augmented by the earlier return of the backward reflected wave to the aorta. Elevated transmission of excessive pulsatile pressure and blood flow promotes the development of cerebral small vessel disease and cognitive dysfunction in aging (Mitchell et al., 2011; Rosano et al., 2013). Indeed, aortic stiffness, as measured by carotid‐femoral pulse wave velocity (cfPWV), predicts declines in cognitive performance, independent of BP, suggesting that stiffness of the conduit arteries that transmit BP and supply blood flow to the brain may be more important than BP alone. Taken together, while these data support the contribution of central arterial hemodynamics to brain aging, these data also suggest that vascular aging represents only a portion of the complex pathophysiology by which cognitive decline and dementia manifest.

Importantly, the pathogenesis of cognitive impairment begins decades prior to the clinical manifestations of reductions in memory, learning, or thinking associated with dementia. However, certain protective behavioral factors may promote resiliency against the development of age‐related neuropathological changes in the brain. Education is an important early life protective risk factor for reducing the dementia risk and delaying the onset of cognitive decline (Hankey, 2018). Education modulates age‐related cognitive decline in part by increasing the cognitive reserve (CR) of the brain (Stern, 2002, 2009). While greater educational attainment does not prevent the development of neuropathology, education increases the “reserve” the brain may utilize to maintain or attenuate declines in cognitive performance (Stern, 2002, 2009). In this regard, an individual with higher educational attainment will be able to accumulate greater amounts of neuropathology (Brickman et al., 2011; Stern, Alexander, Prohovnik, & Mayeux, 1992) (e.g., white matter hyperintensities, *β*‐amyloid deposition, reductions in cerebral blood flow) prior to the manifestation of cognitive decline compared with an individual with fewer years of formal education (Stern, 2002, 2009). Collectively, these studies demonstrate the importance of considering the moderating effects of education on the pathophysiology of cognitive decline. However, cross‐sectional studies linking central artery aging and cognitive performance (Hughes et al., 2018; Mitchell et al., 2011; Pase et al., 2016; Scuteri et al., 2007; Tsao et al., 2013) commonly statistically adjust for years of formal education because education is a well‐known correlate of cognitive performance (Elias, Elias, D'Agostino, Silbershatz, & Wolf, 1997). Despite the critical role of education on cognitive performance, the degree to which education moderates the relation between central artery aging and cognitive performance remains unexplored. Therefore, the purpose of this study was to evaluate the degree to which education moderates the association between central artery aging and cognitive performance. We hypothesized that (1) years of formal education would moderate the relation between central artery aging and cognitive performance; and (2) the relation between central artery aging and cognitive performance would be weaker in individuals with high compared with lower educational attainment in MA/O adults. Support for this would indicate that greater educational attainment may be protective against the adverse effects of central artery aging on cognitive performance.

## 2 METHODS

MA/O adults between the ages of 55 and 85 years with and without atherosclerotic vascular disease (AVD) were recruited through the parent study “Aging, Vascular Disease and Cognition” (Grant number: NIA RO1AG030417‐01A2, PI: David Moser, PhD) from the Iowa City, Iowa community, and the University of Iowa Hospital and Clinics between the years 2008 and 2013. Participants were recruited through flyer and email advertisements to undergo vascular and cognitive testing. Participants were free of major psychiatric or neurological disorders or head injury resulting in loss of consciousness for greater than 30 min. Additionally, participants had no prior history of developmental disorders, systemic illnesses, or neurological disorders that could potentially affect cognition as indicated by detailed health history questionnaire. Female participants were postmenopausal and education groups did not differ by the number of women currently taking hormone replacement therapy during the visit. All participants provided written informed consent to all study protocols that were approved by the Institutional Review Board at the University of Iowa and performed in accordance with ethical standards set by the Declaration of Helsinki.

### 2.1 Measures of education

If education‐related benefits on cognitive performance increase linearly with each successive year of formal education or plateaus at a certain threshold of education remains unclear (Andel, Vigen, Mack, Clark, & Gatz, 2006; Koepsell et al., 2008; Tucker‐Drob, Johnson, & Jones, 2009; Zahodne et al., 2011). To examine this, education was evaluated as both a continuous and dichotomous variable. First, education was used as a continuous variable to test the linear effects of increasing education on the relation between central artery aging and cognition. Years of education (continuous variable) was standardized as the completion of the highest grade or degree of obtainment. Second, education was categorized as ≤HS (i.e., ≤12 years of formal education: HS diploma or less; *n* = 36) or >HS (i.e., ≥13 years of formal education: at least one year of college or greater; *n* = 77) years of education to test the effects of obtaining a certain threshold of formal education. The cutoff of 12 years (i.e., HS diploma) of formal education was determined a priori based on prior literature (Hankey et al., 2018) showing this to be associated with reduced risk of cognitive dysfunction and the hypothesis that this level of educational attainment (i.e., HS diploma) may be more contemporarily relevant provided the high rate (approximately 90%) of HS degree completion in adults over the age of 25 years in the state of Iowa.

### 2.2 Measurements

#### 2.2.1 Aortic stiffness and central blood pressure

Participants reported to the laboratory following an overnight (>8 hr) fasting, having refrained from strenuous physical activity for at least 24 hr prior, and were instructed to hold all vasoactive medication on the morning of vascular testing. After 10 min of supine rest, aortic stiffness and central BP were measured using noninvasive applanation tonometry by pulse wave velocity (PWV) and pulse wave analysis (PWA) (SphygmoCor, AtCor Medical, Inc.). Carotid and femoral pressure waveforms were collected using a noninvasive pressure tonometer. Pressure waveforms were gated to the R‐wave of the ECG to calculate the change in time between the diastolic foot of the pressure waves. The distance between the carotid and femoral pulse sites was measured as the distance between the suprasternal notch (SSN) and the carotid pulse site minus the distance between SSN to the femoral pulse site to account for parallel transmission. cfPWV was calculated as the corrected distance divided by the carotid and femoral foot‐to‐foot delay. Central BP, rather than brachial, was selected a priori because central BP may more closely represent the BP the brain is exposed to compared with brachial BP. Central BP and PP was determined from a radial artery pressure waveform via applanation tonometry and application of a validated transfer function in all participants (Kalil et al., 2016; Pierce et al., 2012). cfPWV and PWA records were collected in triplicate. Records of PWA with an operator index >80% were averaged, consistent with the manufacturer quality control recommendations. Participants (*n* = 6) without at least two records of an operator index <80% were excluded from analyses. Triplicate cfPWV data were averaged. If one value did not meet manufacturer criterion for low variability (<10% of pulse transit time *SD*), then that cfPWV value was omitted and duplicate data were averaged.

#### 2.2.2 Neuropsychological performance

All participants completed cognitive testing administered by a trained research assistant under the supervision of a board‐certified clinical neuropsychologist to assess global cognitive, processing speed, and executive function performance. Global cognitive performance was quantified as the Total Scale Score on the Repeatable Battery for the Assessment of Neuropsychological Status (RBANS) (Moser et al., 2012). Processing speed and executive function were selected *a priori* based on literature demonstrating that these domains of cognition are sensitive to the effects of age‐related increases in large central artery stiffness and BP (Elias et al., 2009; Hajjar, Goldstein, Martin, & Quyyumi, 2016; Mitchell et al., 2011; Poels et al., 2007; Watson et al., 2011). Processing speed and executive function performance were measured by the Stroop Color Word Reading Test as previously described (Hoth, Poppas, Moser, Paul, & Cohen, 2008). Briefly, participants completed the maximal number of correct items (reading words or naming colors) in 45 s from a paper card held by the participant as a measure of processing speed. To minimize multiple comparisons, a composite Z‐score was calculated for the processing speed domain. Stroop interference, which involves discriminating a name of a color from the color the word it is written in, was used to quantify executive function performance. Higher scores indicate better performance on all cognitive tests.

### 2.3 Statistical analysis

All statistical analyses were conducted using IBM SPSS 25.0 software (IBM, Inc.). Two‐tailed Student *t* tests were used to test group differences in subject, vascular and cognitive performance variables. First, multiple linear regression models were used to test the moderating linear effect of education on the relation between the vascular aging variables and cognitive performance in the entire group. Separate linear regression models were tested by first entering the independent predictors (central artery aging variables, age, mean arterial pressure (MAP) [cfPWV only], sex, education [continuous variable], and antihypertensive medication use) of each cognitive domain (global cognitive performance, processing speed Z‐score, and executive function) followed by the relevant education*central artery aging interaction term. An interaction term was calculated by centering each variable of interest (i.e., education [continuous variable], cfPWV, central systolic BP, and central PP) around their mean and then multiplying the centered education variable by the centered vascular variable of interest. The assumption of homoscedasticity was tested in all regression models by visual inspection of scatterplots produced by plotting the regression standardized values versus the regression studentized residuals. There was homoscedasticity in all models.

To test the secondary hypothesis that the relation between central artery aging and cognitive performance would be stronger in those with ≤HS compared with >HS, partial correlations between variables of interest and cognitive performance (adjusted for age, sex, and antihypertensive medication use) were calculated in the >HS and ≤HS groups, separately. Normality was tested using Shapiro–Wilk statistics in all variables included in the present analyses. Central systolic BP, central PP, and cfPWV were square root transformed to normalize the distribution of these variables in the sample. Partial correlations were tested using the normalized variables. Vascular variables of interest that were significantly associated with subdomains of cognitive performance from the partial correlations were entered into the linear regression models to test the interaction between vascular variables of interest and education (categorical variable) in the entire cohort. Separate linear regression models were tested by first entering the independent predictors of each cognitive domain followed by the interaction term. An interaction term was calculated by centering each central artery aging variable of interest around their mean and then multiplying the categorical education variable by the centered vascular variable of interest. Finally, all data are presented as mean ± *SE* and statistical significance was defined as a two‐tailed alpha level of <.05.

## 3 RESULTS

### 3.1 Participant characteristics

One hundred and thirteen MA/O adults completed the study. Participant characteristics are displayed in Table 1. Importantly, the groups did not differ by any CVD risk factors, AVD status, or the use of antihypertensive medication or hormone replacement therapy (Table 1, all *p* > 0.05). Finally, cfPWV (*p* = .75), central systolic BP (*p* = .80), and central PP (*p* = .16) were similar between the education groups (Table 1).

**Table 1:** Subject characteristics

	Entire Cohort (*n* = 113)	>HS (*n* = 77)	≤HS (*n* = 36)	*p*‐value
Age (years)	66.6 ± .7	66.1 ± .8	67.8 ± 1.4	.30
Males/Females	56/57	41/36	15/21	.26
Body mass index (kg/m^2^)	29 ± .5	29.1 ± .6	29.0 ± 1.1	.95
Waist circumference	98 ± 1.5	98 ± 3.3	98 ± 1.6	.91
Hip circumference	107 ± 1.3	104 ± 2.7	108 ± 1.4	.15
Education (years)	14.7 ± .3	16.1 ± .3	11.9 ± .1	<.001*
Triglycerides (mg/dl)	109 ± 5.4	105 ± 6.3	119 ± 10.6	.23
Total cholesterol (mg/dl)	162 ± 3.5	161 ± 4.4	165 ± 5.5	.53
Atherosclerotic vascular disease, *n* (%)	53 (46.9)	33 (43)	20 (56)	.21
Antihypertensive medication, *n* (%)	65 (57.5)	44 (57)	21 (58)	.95
Current hormone replacement use, *n* (%)	7 (6.2)	4 (5)	3 (8)	.94
Blood pressure and vascular outcomes				
Central systolic BP (mmHg)	127 ± 1.6	126 ± 1.8	127 ± 3.2	.80
Central pulse pressure (mmHg)	53 ± 1.5	52 ± 1.7	57 ± 3.1	.16
Carotid‐femoral PWV (m/sec)	10.4 ± .2	10.3 ± .2	10.5 ± .4	.75
Central augmentation index (AIx) adjusted for HR@75 b/min (%)	13 ± .7	12 ± .7	15 ± 1.7	.10
Brachial systolic BP (mmHg)	136 ± 1.6	135 ± 1.9	138 ± 3.1	.46
Brachial pulse pressure (mmHg)	63 ± 1.6	62 ± 1.8	67 ± 3.1	.13
Cognitive performance:				
RBANS total scale score	102 ± 1.1	105 ± 1.4	95 ± 1.9	<.001*
Stroop word reading	90 ± 1.4	92 ± 1.6	84 ± 2.2	<.001*
Stroop color reading	68 ± 1.1	70 ± 1.3	66 ± 1.9	.13
Processing speed Z‐score	−0.07 ± .1	−.01 ± .1	−0.21 ± .1	.11
Stroop interference	35 ± .7	35 ± .9	34 ± 1.2	.28

Data are expressed as mean ± *SE*.

Abbreviations: BP, blood pressure; HS, high school; PWV, pulse wave velocity; RBANS, Repeatable Battery for the Assessment of Neuropsychological Status. Cognitive performance was measured by RBANS Total Scale Score (global cognitive functioning), Stroop Word and Color Reading (processing speed), and Interference (executive function).

* Indicates significance at the *p* < .05 between the >HS and ≤HS groups.

### 3.2 Cognitive performance

Mean cognitive performance data are also presented in Table 1. Global cognitive performance on the RBANS task was weaker in the ≤HS compared with >HS group (*p* < .001). The composite processing speed Z‐score (*p* = .11) did not differ between education groups. However, reading speed was slower on the Stroop Word Reading (*p* = <.001) but not the Stroop Color Naming task (*p* = .13) in the ≤ HS compared with >HS group. Executive function performance on the Stroop Interference trial did not differ between the education groups (*p* = .28).

### 3.3 Moderation of education and vascular aging on cognitive performance in the entire cohort

Tables 2, 3, 4 displays the results of multiple linear regression models calculated to test the interaction between education and vascular variables of interest on global cognitive, processing speed, and executive function performance in separate models. Education (*β* = .24, *p* = .006) and central systolic BP (*β* = −.26, *p* = .007) were associated with processing speed performance in the entire cohort. Education moderated the effect of central systolic BP (education* central systolic BP *β* = .21, *p* = .02) (Table 2). Additionally, the interaction between education* and central PP was significantly associated with processing speed performance (*β* = .22, *p* = .01) when added into the model with education (*β* = .24, *p* = .009) and central PP (*β* = −.20, *p* = .048, Table 3). These data indicate that with increasing years of educational attainment, the negative effect of central systolic BP or PP are attenuated on processing speed performance. Finally, the interaction between education and cfPWV was only marginally associated with processing speed performance (*p* = .09) in the entire cohort (Table S1a).

**Table 2:** Regression analyses of the interaction between education (continuous variable) and central systolic BP on processing speed performance in MA/O adults

Variable	B	*SE*	*β*	*p*‐value
Constant	.57	.62	–	.36
Education	.06	.02	.24	.006*
Age	<.001	.008	<.001	.96
Sex (male)	−.30	.12	−.23	.01*
Antihypertensive medication use	−.16	.11	−.12	.15
Central systolic BP	−.01	.01	−.26	.007*
Education* central systolic BP	.002	.01	.21	.02*

Note

Multiple‐regression modeling including. Overall *F*(6, 112) = 6.31, *p* < .001, *R* = .51, adjusted *R* ^2^ = .26.

Abbreviations: B, unstandardized coefficient; *β*, standardized coefficient.

* *p* < .05.

**Table 3:** Regression analyses of the interaction between education (continuous variable) and central PP on processing speed performance in MA/O adults

Variable	B	*SE*	*β*	*p*‐value
Constant	−.10	.60	–	.87
Education	.06	.02	.24	.009*
Age	−.002	.01	−.02	.85
Sex (male)	−.40	.12	−.30	.001*
Antihypertensive medication use	−.16	.12	−.12	.17
Central PP	−.01	.004	−.20	.048*
Education* central PP	.003	.001	.22	.01*

Note

Multiple‐regression modeling including. Overall *F*(6, 108) = 5.18, *p* < .001, *R* = .48, adjusted *R* ^2^ = .23.

Abbreviations: B, unstandardized coefficient; *β*, standardized coefficient; PP, pulse pressure.

* *p* < .05.

**Table 4:** Regression analyses of the interaction between education (continuous variable) and cfPWV on executive function performance in MA/O adults

Variable	B	*SE*	*β*	*p*‐value
Constant	67.69	8.42	–	<.001*
Education	.44	.24	.16	.07
Age	−.30	.10	−.31	.003*
Sex (male)	−2.55	1.43	−.17	.08
Antihypertensive medication use	−.48	1.31	−.03	.72
cfPWV	−.12	.36	−.03	.75
MAP	−.19	.07	−.27	.005*
Education* cfPWV	.26	.11	.21	.02*

Note

Multiple‐regression modeling including. Overall *F*(7, 109) = 5.45, *p* < .001, *R* = .52, adjusted *R* ^2^ = .27.

Abbreviations: B, unstandardized coefficient; *β*, standardized coefficient; PP, pulse pressure.

* *p* < .05.

In a separate regression model calculated for executive function performance, education* cfPWV was selectively associated with executive function performance (*β* = .21, *p* = .02), while the main effects of MAP (*β* = −.27, *p* = .005) but not education (*β* = .16, *p* = .07) or cfPWV (*β* = −.03, *p* = .75) were associated with executive function alone (Table 4). Consistent with this, the main effects of elevated central systolic BP (*β* = −.29, *p* = .003, Table S1b) and central PP (*β* = −.22, *p* = .03, Table S1c) were independently associated with executive function performance. However, education did not moderate BP or PP in any of these models (Table S1a and b) in the present cohort. Finally, global cognitive performance was not associated with central systolic BP, central PP, or cfPWV nor were their interactions with education statistically significant (data not shown).

### 3.4 Relations between vascular aging and cognitive performance in the education groups

Partial correlations adjusted for age, sex, antihypertensive medication use and MAP (cfPWV only) were performed separately in the ≤HS and >HS groups (Table 5). Greater cfPWV was associated with weaker executive function performance in the ≤HS group (*r* = −.39, *p* = .03), but not in the >HS group (*r* = .15, *p* = .21) (Table 5). Central systolic BP was correlated with processing speed performance in both groups. However, this association was attenuated in the >HS group (*r* = −.25, *p* = .03) compared with ≤HS group (*r* = −.59, *p* < .001). Similarly, higher central systolic BP was associated with weaker executive function performance in the ≤HS (*r* = −.39, *p* = .03) and >HS (*r* = −.32, *p* = .006) groups. Greater central PP was selectively associated with slower processing speed (*r* = −.62, *p* < .001) in the ≤HS group only. However, the magnitude of the partial correlations did not differ between education groups (Table S2a and b) as evidenced by the nonsignificant interaction terms except in the domain of executive function. In this domain, the relation between cfPWV and executive function performance was significantly attenuated in the group with >HS compared with ≤HS independent of MAP (Table 6). Finally, global cognitive performance was not correlated with any vascular variables of interest in either group.

**Table 5:** Partial correlation coefficients between aortic stiffness and blood pressure variables with cognitive performance outcomes among MA/O individuals separated by education group

	>High School (*n* = 77)			≤High School (*n* = 36)		
	Global cognitive	Processing speed	Executive function	Global cognitive	Processing speed	Executive function
CfPWV	.09 (.47)	.07 (.57)	.15 (.21)	.04 (.81)	−.14 (.43)	−.39 (.03)* ψ
Central systolic BP	−.16 (.17)	−.25 (.03)*	−.32 (.006)*	.10 (.55)	−.59 (<.001)*	−.39 (.03)*
Central pulse pressure	−.21 (.08)	−.11 (.38)	−.21 (.08)	.25 (.14)	−.62 (<.001)*	−.28 (.14)

Note

Partial correlations adjusted for age, sex, medication, and mean arterial pressure (cfPWV only) among MA/Oadults with > HS and ≤ HS diplomas. Abbreviations: CfPWV, carotid‐femoral pulse wave velocity; BP, blood pressure.

* Indicates *p* < .05; ψ indicates significant differences in correlational coefficients between group as determined by multiple linear regression

**Table 6:** Regression analysis of the interaction between education group (i.e >HS and ≤HS) and cfPWV on executive function performance in MA/O adults

Variable	B	*SE*	*β*	*p*‐value
Constant	80.95	9.27	–	<.001*
Education group	−11.96	6.51	−.76	.07
Age	−0.26	0.10	−.28	.008*
Sex (male)	−2.10	1.45	−.14	.15
Antihypertensive medication use	−0.52	1.34	−.04	.70
cfPWV	−0.92	0.50	−.27	.07
MAP	−0.21	0.07	−.29	.003*
Education group* cfPWV	1.33	0.62	.92	.03*

Note

Multiple‐regression modeling including. Overall *F*(7, 109) = 4.77, *p* < .001, *R* = .50, adjusted *R* ^2^ = .25.

Abbreviations: B, unstandardized coefficient; *β*, standardized coefficient; cfPWV, carotid femoral pulse wave velocity; MAP, mean arterial pressure.

* *p* < .05.

## 4 DISCUSSION

The primary objective of this study was to determine the degree to which education moderates the relation between central artery aging and cognitive performance in MA/O adults. To determine this, education was treated as a continuous and dichotomous variable to test the linear and threshold effects of years of formal educational completion, respectively. We demonstrated that the main effects of central artery aging, including central systolic BP and PP, but not cfPWV, were associated with lower performance on processing speed and executive function performance in the present cohort. Second, education, defined as a continuous variable, significantly attenuated the association between central artery aging and cognitive performance. Third, the relation between cfPWV and executive function was attenuated in individuals with >HS compared with ≤HS in this study. In contrast, higher central systolic BP and PP were associated with slower processing speed and weaker executive function performance in MA/O adults with ≤HS and  >HS groups. However, these effects (i.e., correlational coefficients) did not differ between education groups as demonstrated by the nonsignificant interaction terms in the >HS versus ≤HS groups. Taken together, these data support our hypothesis that education moderates the adverse effects of central artery aging on cognitive performance in MA/O adults.

Increasing evidence supports the contribution of age‐related changes in the large central artery stiffness‐related hemodynamics and central BP in the complex pathophysiology of cognitive decline (Mitchell et al., 2011; Pase et al., 2016; Scuteri et al., 2007). Stiffening of the large central arteries reduces the mismatch in the impedance gradient between the low (i.e., central elastic) and the high impedance (i.e., peripheral muscular) arteries (Mitchell et al., 2011). Increased impedance matching augments the transmission of elevated pulsatile energy and blood flow to the vulnerable high flow, low impedance cerebral microvasculature. Chronic transmission of elevated pulsatile pressure and blood flow are hypothesized to promote cerebrovascular remodeling and augmented cerebrovascular resistance to limit the penetration of excessive pressure to the downstream neurovascular units (Mitchell et al., 2011; Tarumi et al., 2014). However, remodeling of the cerebrovasculature occurs at the expense of cerebral blood flow regulation (DuBose et al., 2018; Jefferson et al., 2018), thereby increasing the susceptibility of the cerebral microstructure to hypoperfusion (Tarumi et al., 2014; Tarumi, Shah, Tanaka, & Haley, 2011). Indeed, elevated aortic stiffness, central BP, and PP are independent risk factors for the development of cerebral small vessel disease (Rosano et al., 2013; Shrestha et al., 2009; Webb et al., 2012) with one standard deviation increase in cfPWV, equivalent to 1.2 years of brain aging 35 and approximately 60%–70% increased risk for subcortical infarcts (Mitchell et al., 2011) in MA/O adults with CVD risk factors. In the present study, education defined as a continuous but not dichotomous variable, significantly moderated the relation between central systolic BP and PP on processing speed. Processing speed is the initial domain of cognition to decline with advancing age and is hypothesized to mediate reductions in subsequent domains of cognitive performance including executive function (Salthouse, 1992). Therefore, it is possible that greater educational attainment may attenuate the detrimental effects of BP and PP on processing speed and subsequently slowing declines in other domains of cognition. However, in the present study, both threshold (>HS) and continuous effects of education on the relation between cfPWV and executive function were detected despite the lack of group differences in cfPWV and executive function performance. These data support prior studies that demonstrate the detrimental effects of age‐related increases in aortic stiffness are strongest on domains of cognition associated with subcortical white matter including executive functioning in MA/O adults (Mitchell et al., 2011). Furthermore, these data extend prior findings by demonstrating that education moderates this relation particularly in individuals with more years of education by weakening the relation between cfPWV and executive function performance. Taken together, these data suggest that education may not only be an important covariate of cognitive performance but also a critical moderator of the effects of central artery aging on cognition.

Numerous studies demonstrate that greater educational attainment is protective against age‐related cognitive decline by increasing the “reserve” the brain may utilize to maintain or attenuate changes in cognition (Gordon et al., 2008; Hankey, 2018; Stern, 2012; Stern et al., 1992). In this regard, greater educational attainment may be protective by increasing the brain's CR. CR is defined as an active process developed from experiential resources, such as education or occupational complexity. CR enables the brain to cope with the development of neuropathology to maintain or attenuate declines in brain function and cognitive performance using preexisting cognitive processing or by recruiting compensatory neural pathways (Stern, 2012). The CR hypothesis was initially proposed to explain discrepancies between a brain's neuropathological load and observed cognition. In support of this, Stern et al., (1992) previously demonstrated that individuals with the greatest educational attainment had greater volumes of white matter lesions and reductions in basal temporal‐parietal cerebral blood flow, despite being matched for clinical Alzheimer’s disease severity. Therefore, in individuals with greater educational attainment, age‐related decline will be slower until the threshold for neuropathology sufficient to induce cognitive dysfunction is reached, at which point, the rate of decline will be greater in those with high CR (Andel et al., 2006). This suggests that greater educational attainment may not prevent cognitive impairment or dementia, but rather may be associated with an increased overall quality of life by reducing the number of years an individual is impaired.

The precise mechanism by which education moderates cognitive performance with aging remains unclear. Greater educational attainment is hypothesized to increase the number of neural connections (i.e., neural reserve) or provide more alternate neural pathways that may be used if primary neural pathways become damaged (i.e., neural compensation) (Stern, 2012). However, greater educational attainment may also alter memory encoding processes in the brain leading to “richer” memories being formed or enhanced memory retrieval associated with optimal organizational structure or reconstruction of stored memories (Salthouse, 2003). While it remains unclear the primary mechanism by which education moderates the relation between neuropathology and cognition, these hypotheses support the idea that the brain actively attempts to cope with the development of neuropathology to delay or attenuate cognitive decline and impairment in aging. It is possible that the effects of aortic stiffness and central BP components on cognition demonstrated in the present study are mediated in part by the development of cerebral small vessel disease (Abraham et al., 2016; Aribisala et al., 2014; Dufouil et al., 2001) and attenuated by greater educational attainment. Furthermore, education moderates the effects of cerebral white matter lesions on cognition (Mortamais et al., 2014). Results of the present study support this hypothesis by demonstrating that the effects of vascular aging, including central systolic BP and PP, had a stronger negative association on cognition in individuals with fewer years of education. Taken together, the present study adds to the current body of knowledge that education may moderate the effects of vascular aging on cognitive performance in MA/O adults and therefore should be at least considered as a moderator in future studies.

This study should be interpreted in the context of several limitations. First, this study did not assess alternative moderators (i.e., occupational complexity, leisure time activities or physical activity) of CR (Stern, Albert, Tang, & Tsai, 1999; Stern et al., 1994). This is important because education is primarily achieved in the first several decades of life and ending before midlife, an important “sensitive” period for vascular aging. Therefore, the degree of occupational complexity or cognitively stimulating leisure time activities including physical activity or reading frequency (Sörman, Ljungberg, & Reading, 2018) also may be moderators of CR on cognitive aging because they occur with greater frequency during midlife compared with education. Prior studies have indicated that higher levels of educational attainment, occupational complexity, and physical activity may reduce the risk of cognitive impairment by 46% (Chan et al., 2018). Second, education treated as a continuous and dichotomous variable was defined as individuals who had completed 12 years or less of formal education. This threshold was selected based on data in which 92% of individuals over the age of 25 have received at least a HS level diploma in the state of Iowa. The educational threshold sufficient for being cognitively protective is unclear with some groups defining educational attainment by more conservative cutoffs of <8 years of formal education (Stern et al., 1999; Zahodne, Stern, & Manly, 2015) or contemporarily relevant criterion of <12 years (Andel et al., 2006; Zahodne et al., 2011) similar to the present study. However, it is important to note that differences in average global cognitive performance and the relation between vascular risk factors and cognitive performance were detectable in the present study with the groups being separated by attainment of a HS diploma. These cutoffs may be more relevant in developed countries because approximately 84% of American students complete a HS diploma (NCES, 2017). Third, socioeconomic status (SES) was not measured in the present study and may influence educational attainment, cardiovascular health and cognition (Climie et al., 2019; Greenfield & Moorman, 2018). Recent data indicate that SES, both at the individual and neighborhood level, is associated with increased central artery stiffness in MA/O adults (Climie et al., 2019). Therefore, we cannot determine whether education, measured in the present study, is a surrogate for individual SES or if the effects of education on cognition demonstrated in the present study are independent of SES. Fourth, the current study did not measure functional connectively or white matter microstructure to test the mechanism by which education moderated the effects of vascular aging on cognitive performance. Future studies should be conducted to explore neural compensation and neural reserve as potential mechanisms by which education moderates the vascular contribution to cognitive aging. Fifth, the SphygmoCor device derives central aortic BP by calibrating the radial waveform to the brachial systolic and diastolic BP measured by oscillometric BP cuff. A recent meta‐analysis indicates that this calibration method underestimates central BPs to a greater extent compared with the standard method (−7.8 vs. 3.0 mmHg) which uses the mean and diastolic BP. Therefore, while the reported central BP values may underestimate actual central BP values, the purpose of the study was to evaluate the role of education on central BP across a continuous range of central BPs rather than a dichotomous BP value. Finally, the cross‐sectional design of this study does not allow for the determination of causality between education and cognitive function.

In summary, the results of this study demonstrate that effects of central artery aging, including central systolic BP, PP and aortic stiffness, on processing speed and executive function performance are attenuated in individuals with greater education attainment. These data suggest that education is an important moderator of the degree to which age‐related central artery aging, including elevated central systolic BP and PP, impact cognitive performance. In this regard, MA/O adults with fewer years of education may be more sensitive to the detrimental effects of age‐related increases in central systolic BP, PP and aortic stiffness on cognitive performance. However, further studies are needed to explore the degree to which other moderators of CR, such as occupational complexity, attenuate the relation between vascular aging and cognitive performance in aging.

## CONFLICT OF INTEREST

The authors have no disclosures to report.

## AUTHORS CONTRIBUTIONS

D.J.M and L.E.D. conceived and designed the study; D.J.M and E.H. performed experiments; D.J.M., L.E.D., and J.G.F. analyzed the data; D.J.M, L.E.D, J.G.F., and G.L.P interpreted the results of the experiments; L.E.D. prepared the figures; L.E.D. drafted manuscript; D.J.M, G.L.P, L.E.D., E.H., and J.G.F. edited and revised manuscript; D.J.M., G.L.P., L.E.D., E.H., and J.G.F. approved final version of the manuscript.
