# Supplementary material for: Education moderates the effects of large central artery aging on cognitive performance in middle‐aged and older adults
Source: Physiol Rep. 2019 Dec 12;7(23):e14291. doi: 10.14814/phy2.14291 (PMC6908737; doi:10.14814/phy2.14291)
Supplement: Supplementary file 1 [file PHY2-7-e14291-s001.docx]

**Supplemental Tables: Education as a Continuous Variable:**

**Supplemental Table 1A:** Regression analysis of the interaction between education (continuous variable) and cfPWV on processing speed performance in MA/O adults.

| Variable | B | SE | β | ***p-value*** |
| --- | --- | --- | --- | --- |
| Constant | 1.39 | 0.75 | - | 0.06 |
| Education | 0.06 | 0.02 | 0.24 | **0.008*** |
| Age | -0.01 | 0.01 | -0.09 | 0.38 |
| Sex | -0.17 | 0.13 | -0.13 | 0.18 |
| Anti-hypertensive medication use | -0.22 | 0.12 | -0.17 | 0.06 |
| cfPWV | 0.01 | 0.03 | 0.03 | 0.76 |
| MAP | -0.02 | 0.01 | -0.31 | **0.002*** |
| Education *cfPWV | 0.02 | 0.01 | 0.15 | 0.09 |

Multiple-regression modeling including B, unstandardized coefficient; β, standardized coefficient. cfPWV, carotid-femoral pulse wave velocity.

Overall F(7,112)= 4.87, P<0.001, R=0.50, adjusted R^2^= 0.25. * indicates p<0.05.

**Supplemental Table 1B:** Regression analysis of the interaction between education (continuous variable) and central systolic BP on executive function performance in MA/O adults.

| Variable | B | SE | β | ***p-value*** |
| --- | --- | --- | --- | --- |
| Constant | 60.02 | 7.15 | - | **<0.001*** |
| Education | 0.53 | 0.25 | 0.19 | **0.04*** |
| Age | -0.22 | 0.09 | -0.24 | **0.02*** |
| Sex | -3.65 | 1.39 | -0.25 | **0.01*** |
| Anti-hypertensive medication use | 0.10 | 1.30 | 0.01 | 0.94 |
| Central systolic BP | -0.13 | 0.04 | -0.29 | **0**.**003*** |
| Education * central systolic BP | 0.01 | 0.02 | 0.09 | 0.32 |

Multiple-regression modeling including B, unstandardized coefficient; β, standardized coefficient. BP, blood pressure.

Overall F(6,109)= 5.66, P<0.001, R=0.50, adjusted R^2^= 0.20. * indicates p<0.05.

**Supplemental Table 1C:** Regression analysis of the interaction between education (continuous variable) and central PP on executive function performance in MA/O adults.

| Variable | B | SE | β | ***p-value*** |
| --- | --- | --- | --- | --- |
| Constant | 51.56 | 6.90 | - | **<0.001*** |
| Education | 0.54 | 0.26 | 0.19 | **0.041*** |
| Age | -0.26 | 0.10 | -0.27 | **0.008*** |
| Sex | -4.49 | 1.44 | -0.30 | **0.002*** |
| Anti-hypertensive medication use | 0.33 | 1.37 | 0.02 | 0.81 |
| Central PP | -0.11 | 0.05 | -0.22 | **0.03*** |
| Education * central PP | 0.01 | 0.02 | 0.01 | 0.91 |

Multiple-regression modeling including B, unstandardized coefficient; β, standardized coefficient. PP, pulse pressure.

Overall F(6,105)= 4.47, P<0.001, R=0.46, adjusted R^2^= 0.21. * indicates p<0.05.

**Supplemental Tables: Education as a Dichotomous Variable:**

**Supplemental Table 2A.** Regression analysis of the interaction between education group (i.e., >HS and ≤HS) and central systolic BP on processing speed performance in MA/O adults.

Multiple-regression modeling including B, unstandardized coefficient; β, standardized coefficient. BP, blood pressure.

Overall F(6, 109)= 4.26, P=0.001, R=0.44, adjusted R^2^= 0.20. * indicates p<0.05.

| Variable | B | SE | β | ***p-value*** |
| --- | --- | --- | --- | --- |
| Constant | 1.81 | 0.82 | - | **0.03*** |
| Education group | -0.68 | 0.92 | -0.48 | 0.46 |
| Age | 0.004 | 0.01 | 0.05 | 0.65 |
| Sex | -0.24 | 0.13 | -0.20 | 0.06 |
| Anti-hypertensive medication use | 0.15 | 0.12 | -0.11 | 0.21 |
| Central Systolic BP | -0.02 | 0.01 | -0.43 | **0.004*** |
| Education group *central systolic BP | 0.01 | 0.01 | 0.67 | 0.31 |

**Supplemental Table 2B.** Regression analysis of the interaction between education group (i.e., >HS and ≤HS) and central PP on processing speed performance in MA/O adults.

| Variable | B | SE | β | ***p-value*** |
| --- | --- | --- | --- | --- |
| Constant | 0.87 | 0.63 | - | 0.170 |
| Education group | -0.43 | 0.45 | -0.30 | 0.34 |
| Age | 0.002 | 0.01 | 0.02 | 0.86 |
| Sex | -0.36 | 0.13 | -0.27 | **0.005*** |
| Anti-hypertensive medication use | -0.16 | 0.12 | -0.12 | 0.20 |
| Central PP | -0.02 | 0.01 | -0.41 | **0.01*** |
| Education Group * central PP | 0.01 | 0.01 | 0.48 | 0.14 |

Multiple-regression modeling including. B, unstandardized coefficient; β, standardized coefficient. PP, pulse pressure.

Overall F(6, 108)= 3.43, P=0.004, R=0.41, adjusted R^2^= 0.17. * indicates p<0.05.

**Supplemental Table 2C.** Regression analysis of the interaction between education group (i.e., >HS and ≤HS) and central systolic BP on executive function in MA/O adults.

| Variable | B | SE | β | ***p-value*** |
| --- | --- | --- | --- | --- |
| Constant | 65.93 | 9.89 | - | **<0.001*** |
| Education group | 0.44 | 11.16 | 0.03 | 0.97 |
| Age | -0.20 | 0.09 | -0.21 | **0.04*** |
| Sex | -3.03 | 1.43 | -0.21 | **0.04*** |
| Anti-hypertensive medication use | 0.26 | 1.32 | 0.02 | 0.84 |
| Central Systolic BP | -0.14 | 0.07 | -0.31 | **0.04*** |
| Education group * central systolic BP | 0.01 | 0.09 | 0.09 | 0.90 |

Multiple-regression modeling including B, unstandardized coefficient; β, standardized coefficient. BP, blood pressure.

Overall F(6, 109)= 4.83, P<0.001, R=0.47, adjusted R^2^= 0.22. * indicates p<0.05.
